# MAIT cells: new guardians of the liver

**DOI:** 10.1038/cti.2017.5

**Published:** 2017-02-24

**Authors:** Ayako Kurioka, Lucy J Walker, Paul Klenerman, Christian B Willberg

**Correction to:** *Clinical & Translational Immunology* (2016) **5**, e98; doi:10.1038/cti.2016.51; published online 19 August 2016

This article was originally published under a CC BY-NC-ND 4.0 license, but has now been made available under a CC BY 4.0 license. The PDF and HTML versions of the paper have been modified accordingly.

